# Activation and lineage specific Lag3 expression dynamics in T cells

**DOI:** 10.1093/jimmun/vkag039

**Published:** 2026-03-28

**Authors:** Chaimae Khaled, Sohee Kim, Dongkyun Kim, Booki Min

**Affiliations:** Department of Microbiology and Immunology, Northwestern University Feinberg School of Medicine, Chicago, IL, United States; Department of Microbiology and Immunology, Northwestern University Feinberg School of Medicine, Chicago, IL, United States; Department of Microbiology and Immunology, Northwestern University Feinberg School of Medicine, Chicago, IL, United States; Center for Human Immunobiology, Northwestern University Feinberg School of Medicine, Chicago, IL, United States; Department of Microbiology and Immunology, Northwestern University Feinberg School of Medicine, Chicago, IL, United States; Center for Human Immunobiology, Northwestern University Feinberg School of Medicine, Chicago, IL, United States

**Keywords:** cycling, dynamics, Lag3, T cells

## Abstract

Co-inhibitory receptors are essential checkpoints to restrain excessive T cell activation. While PD1 and CTLA4 have been extensively studied, the biology of lymphocyte activation gene 3 (Lag3), an emerging target for checkpoint blockade immunotherapy, remains less understood. In this study, we show that Lag3, though largely intracellular in resting T cells, exhibits unexpected dynamic cycling even without stimulation. The Lag3 cycling are influenced by lineage and differentiation status, particularly within memory and regulatory T cell subsets. Moreover, Lag3-like cycling patterns were also observed in other co-receptors with stimulatory and inhibitory properties. Mechanistically, endosomal trafficking is important for Lag3 cycling, whereas the cytoplasmic domain of Lag3 appears to be largely dispensable for this process. T cells engaging in Lag3 cycling exhibit a survival and proliferation advantage in both lymphpenic and lymphoreplete conditions. Together, these findings establish Lag3 cycling as a regulated and functionally important process, providing new insights into the biology of Lag3 in T cells.

## Introduction

Lymphocyte activation gene 3 (Lag3) is an activation-induced co-inhibitory receptor predominantly expressed on T cells with exhausted and regulatory features.^1^^,^^2^ Most studies to date have focused on its role in dampening T cell responses in cancer and chronic viral infections.^3^ Recent clinical studies demonstrated that Lag3-blockade, particularly in combination with PD1 inhibition, holds significant promise for restoring anti-tumor T cell function.^4^^,^^5^ Despite these advances, the intracellular trafficking, signaling mechanisms, and precise molecular functions of Lag3 remain poorly defined.

PD1 and CTLA4 were the first co-inhibitory receptors to be extensively characterized and targeted in immunotherapy.^6^ More recently, Lag3 has emerged as the next major immune checkpoint receptor with therapeutic potential.^7^ These receptors share overlapping immunosuppressive functions, including the inhibition of T cell proliferation and the suppression of cytokine production.^8^^,^^9^ Mechanistically, PD1 engagement induces phosphorylation of two conserved tyrosine residues within its cytoplasmic domain—the immunoreceptor tyrosine-based inhibitory motif (ITIM) and the immunoreceptor tyrosine-based switch motif (ITSM). These phosphotyrosines recruit SHIP1 and SHIP2 phosphatases, which in turn suppress key TCR and CD28 co-stimulatory pathways, including ZAP70 and PI3K.^10^ PD1 surface expression and stability are further regulated by glycosylation of its extracellular IgV domain by fucosyltransferase 8 (Fut8) that modulate surface expression and by the thymocyte selection-associated high mobility group box protein (Tox), a master regulator of T cell exhaustion, which facilitate PD1 recycling through direct interaction with PD1 cytoplasmic domain.^11^ CTLA4 contains a conserved YVKM that functions as a regulatory hub for its trafficking and signaling. When phosphorylated, the motif serves as binding site for PI3K,^12^ whereas in its non-phosphorylated state, it serves as a binding site for the clathrin adaptor complex AP-2 to promote internalization into endosomes.^13^ Therefore, the phosphorylation status of the motif determines CTLA4 surface retention. Internalized CTLA4 can be recycled back to the cell surface through the LPS-responsive beige-like anchor protein (LRBA).^14^ Notably, CTLA4 undergoes constitutive cycling in regulatory T (Treg) cells,^15^ a process proposed to be essential for the uptake and degradation of CD80/86 ligands, thereby mediating immune suppressive functions of Treg cells.^16^ Supporting the model, LRBA mutation reduces surface CTLA4 expression in T cells and is associated with autoimmune lymphoproliferative conditions.^17^

Previous studies examining the intracellular trafficking of Lag3 have demonstrated that, in unstimulated T cells, Lag3 is predominantly localized within late endosomes and lysosomes, and upon activation, it rapidly translocates to the cell surface.^18^^,^^19^ Quantification of Lag3^+^ cells in naïve mouse splenocytes revealed that only ∼2% of total CD4 and CD8 T cells express Lag3 on the surface.^20^ These Lag3^+^ cells were further characterized as CD44^high^ and actively dividing, suggesting that a small subset of Lag3^+^ T cells in naïve mice represent proliferating memory phenotype cells.^20^ However, the process governing Lag3 expression and regulation in T cells remains largely unknown.

In this study, we demonstrate that Lag3 undergoes spontaneous cycling in unstimulated T cells, with CD44^high^ memory/effector and regulatory subsets exhibiting markedly greater cycling activity compared to their CD44^low^ and conventional counterparts. Upon activation, Lag3 cycling dynamics are substantially enhanced, highlighting its regulation in response to cellular stimulation. Interestingly, enhanced cycling in memory and regulatory subsets was also observed for other coreceptors with stimulatory or inhibitory properties, suggesting that this may represent a general feature contributing to cellular function. At the mechanistic level, we found the contributions of endosomal trafficking in mediating this process, while the Lag3 cytoplasmic domain was found to be largely dispensable. We also demonstrated that cells engaged in Lag3 cycling acquire a physiological survival and proliferation advantage under lymphopenic and steady-state conditions. Taken together, these findings establish Lag3 cycling as a regulated and functionally important process, offering new insights into cellular regulation of Lag3 in T cells.

## Materials and methods

### Animals

Foxp3^DTR^ (B6.129(Cg)-Foxp3tm3(DTR/GFP)Ayr/J, no. 016958) and germ-line *Lag3*^−/−^ mice (B6.129S2-Lag3tm1Doi/J, no. 026644) were purchased from the Jackson Laboratory (Bar Harbor, Maine, USA). The generation of Lag3^OE^ mice was commissioned by Biocytogen (Beijing, China). In brief, a targeting vector containing the CAG promoter, a floxed stop cassette, Lag3 cDNA, the woodchuck hepatitis virus post-transcriptional regulatory element, and bovine growth hormone polyadenylation signal, was inserted into the *Rosa26* locus. These mice were then crossed with CD4^Cre^ transgenic mice (B6.Cg-Tg(Cd4-cre)1Cwi/BfluJ, no. 022071, Jackson Laboratory) to generate T cell-specific Lag3-overexpressing mice. Mice were used at between 6 and 12 wk of age. Both male and female mice were used. All the mouse protocols were approved by the Institutional Animal Care and Use Committee (IACUC) of the Northwestern University. The mice were maintained under specific pathogen-free conditions at the Northwestern University Feinberg School of Medicine.

### Cycling assay

Lymph nodes were harvested from mice and processed into a single cell suspension using a 40 µM cell strainer. Live cells were resuspended at a concentration of 5 × 10^6^ cell/ml in cPRMI and with 6 µg/ml of PE-conjugated anti-Lag3 (C9B7W) monoclonal antibody (BioLegend, San Diego, California, USA). A total of 200 µl of cell suspension was plated per well in a 96-well round-bottom plate and incubated at 37 °C in an incubator with 5% CO_2_. Cells were collected at 1-, 2-, and 4-h postincubation, washed and stained with conjugated antibodies against other surface markers and then fixed in 4% PFA for 10 min at room temperature. Time 0 samples were kept on ice for 20 min to stain surface Lag3 then washed and stained with conjugated antibodies against surface markers and fixed as above. In some experiments, cells were treated with 5 μg/ml of anti-I-A^b^ antibody (Y3P) for 15 min on ice prior to undergoing the Lag3 cycling assay. For in vivo Lag3 cycling assay, B6 mice were injected intravenously with 100 µl of 5 µg/ml of PE-conjugated anti-Lag3 monoclonal antibody. Mice were sacrificed at 2- or 4-h postinjection. Spleens were harvested, processed into a single cell suspension, and stained with antibodies against surface markers. Cells were then fixed and acquired as above. In some experiments, pronase was used to remove surface molecules. Pronase was purchased from Millipore Sigma (St. Louis, MO) and prepared according to the manufacturer’s instructions. Cells (10 × 10^6^ cells/ml) were incubated with pronase in cRPMI for 15 min at 37 °C in the incubator. After incubation, cells were washed three times with cRPMI and then used for subsequent experiments. For experiments assessing the role of endosomal recycling, pronase treated cells were incubated with anti-Lag3 antibody for 1 or 2 h at either 37 °C or 16 °C on a heating block.

### In vitro activation

Naive (CD25^−^ CD44^low^) CD4 and CD8 T cells were sorted using a FACSMelody cell sorter (BD Biosciences, San Jose, CA). Sorted cells were seeded into plates precoated with 2 µg/ml anti-CD3 (2C11) and anti-CD28 (37.51) antibodies. After 48 h cells were collected, washed and reseeded in fresh cRPMI supplemented with 30U/ml of recombinant human IL2 (obtained from the Biometric Research Program, National Cancer Institute) to allow for resting. For in vitro exhaustion assay, FACS-sorted naive (CD44^low^) CD8 T cells were seeded into plates precoated with anti-CD3 (5 µg/ml) and anti-CD28 (2 µg/ml) antibodies. After 3 d of stimulation, chronically stimulated cells were passaged every 2 d onto freshly coated plates containing anti-CD3 (5 µg/ml) alone. Acutely stimulated cells were maintained in media supplemented with 100U/ml recombinant human IL-2 and passaged every 2 d without additional plate-bound stimulation. Chronic and acutely stimulated cells were collected at day 8 and used for cycling assay.

### Flow cytometry

Flow cytometric analysis was performed with anti-CD4 (RM4-5), anti-CD44 (IM7), anti-CD8 (53-6.7), anti-CD25 (PC61.5), anti-CD45 (I3/2.3), anti-PD1 (J43), anti-Tim3 (RMT3-23), anti-CTLA4 (UC10-4B9), anti-CD127 (A7R34), anti-CD122 (TM-β1), anti-CD40L (MR1), anti-CD137 (17B5), anti-Ki67 (11F6), and anti-Bcl2 (10C4) antibodies (purchased from Biolegend). Samples were acquired on a BD Symphony A1 flow cytometer (BD Biosciences), and data were analyzed using FlowJo v10 software (TreeStar, Ashland, Oregon, USA).

### Experimental autoimmune encephalomyelitis (EAE) induction

EAE was induced by subcutaneous immunization with a 1:1 emulsion of MOG_35_-_55_ peptide (300 μg; BioSynthesis, Lewisville, Texas, USA) in complete Freund’s adjuvant (CFA) containing 5 mg/mL Mycobacterium tuberculosis H37Ra (Difco, Detroit, Michigan, USA). Mice received 200 ng of pertussis toxin (Millipore Sigma) via intraperitoneal injection on days 0 and 2 postimmunization. Mice were monitored daily for clinical signs of EAE and sacrificed at peak disease. Brains and spinal cords were harvested and processed into single-cell suspensions via chemical dissociation, mechanical straining, and Percoll gradient centrifugation, as previously described.^21^ Cells were then subjected to the Lag3 cycling assay for 2 and 4 h as detailed above.

### Irradiation and homeostatic proliferation

Mice were sublethally irradiated with 600 cGy and injected intravenously with 0.5 × 10^6^ CFSE-labeled Lag3^+^ cycling naive CD4^+^ and CD8^+^ T cells, or with CFSE-labeled Lag3^+^ non-cycling naive CD4^+^ and CD8^+^ T cells. Cycling and non-cycling populations that were identified from incubating cells with PE-conjugated anti-Lag3 antibody for one hour. The recipients were sacrificed 14 days after the transfer and homeostatic proliferation of the donor T cells was determined by flow cytometry.

### Statistical analysis

Statistical significance was determined using the Mann–Whitney *U* test or 2-way repeated-measures ANOVA with Geisser–Greenhouse correction, followed by Tukey’s post hoc test for pair wise comparisons. All analyses were conducted using a Prism software (GraphPad, San Diego, California, USA). A *P* value <0.05 was considered statistically significant.

## Results

### T cells cycle Lag3 at steady state condition

While co-inhibitory receptors are minimally expressed in most resting T cells, certain T cell subsets, especially Foxp3^+^ Treg cells, constitutively express a battery of the receptors, including CTLA4 and Lag3.^22^ Yet, the surface expression is not evident, and instead its cellular location is largely limited to the intracellular vesicles.^23^ Campbell and colleagues recently measured the cycling pool of CTLA4 by labeling live cells with fluorochrome-conjugated anti-CTLA4 Ab at 37 °C and observed active CTLA4 cycling in CD44^high^ effector Treg cells.^24^ Since Lag3 expression resembles that of CTLA4, we sought out to test whether Lag3 similarly cycles in T cells. Lymph node cells were incubated with PE-conjugated anti-Lag3 Ab at 37 °C, harvested after 1, 2, and 4 hr of incubation, and examined for PE labeling. For the 0-hr time point cells were incubated with the Ab at 4 °C. The proportions of PE-labeled cells continuously increased over time in both CD4 and CD8 T cells (Fig. 1A). The staining intensity measured by geometric mean fluorescence intensity (gMFI) showed gradual increase over time.

**Figure 1:**
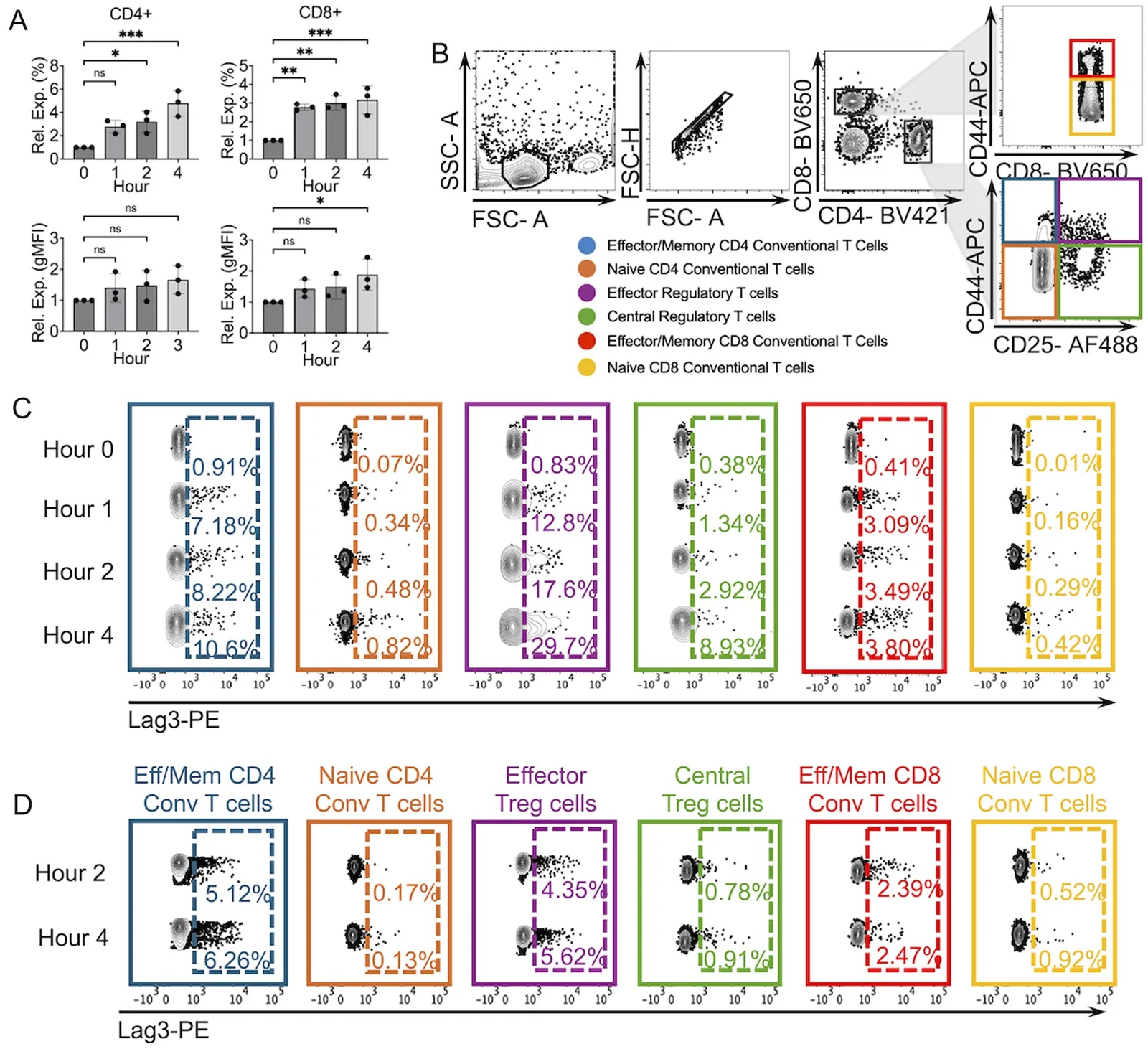
Lag3 Cycling in T cells subsets at resting state. (A) Relative expression of Lag3 cycling in CD4 and CD8 T cells over a 4-hour time course. Lymph node cells were incubated with PE conjugated anti-Lag3 antibody at 37°C and collected at 0, 1, 2, and 4 h. Data represent relative changes in Lag3 frequency and gMFI for CD4 and CD8 T cells, normalized to the initial time point (Hour 0). Data are shown as the mean ± SEM. *P <0.05; **P<0.01; ***P<0.001. (B) Gating strategy used to identify T cell subsets. Cells were gated sequentially based on lymphocytes (FSC vs SSC), single-cell discrimination (FSC-A vs FSC-H), and CD4 vs CD8 populations. CD4 and CD8 T cells were further divided into CD4^+^CD44^high^ CD25^−^ for effector/memory CD4 conventional T cells, CD4^+^CD44^low^CD25^−^ for naive CD4 conventional T cells, CD4^+^CD44^high^CD25^+^ for effector regulatory T cells, CD4^+^CD44^low^CD25^+^ for central regulatory T cells, CD8^+^CD44^high^ for effector/memory CD8 conventional T cells and CD8^+^CD44^low^for naive CD8conventional T cells. (C) Representative dot plots of each T cell subset at each time point (hours 0, 1, 2, and 4). Percentages denote Lag3^+^ cells within the gate. (D) Representative dot plots of splenocytes after in vivo labeling of Lag3 cycling for each subset 2 and 4 hours after injection with PE conjugated anti-Lag3 antibody. Data from in vitro experiments shown are a representative of three independent experiments. In vivo labeling experiments were from two independent animals.

Since CTLA4 cycling was most pronounced in effector Treg cells,^24^ it is possible that Lag3 cycling also differs depending on the cellular differentiation status (ie naive vs memory phenotype). Lag3 cycling was thus compared between CD4 and CD8 T cells expressing CD25 and/or CD44: CD4^+^CD25^−^CD44^high^ effector/memory CD4 conventional, CD4^+^CD25^−^CD44^low^ naive CD4 conventional, CD4^+^CD25^+^CD44^high^ effector regulatory, CD4^+^CD25^+^CD44^low^ central regulatory, CD8^+^CD44^high^ effector/memory CD8 conventional, and CD8^+^CD44^low^ naïve CD8 conventional T cells (Fig. 1B). Lag3 cycling was most evident in CD44^high^ memory phenotype T cells compared to their naive counterparts (Fig. 1C and S1A). Among the CD44^high^ phenotype cells, effector Treg cells exhibited the most striking Lag3 cycling (Fig. 1C and S1A). Repeating the experiments using germline Lag3^−/−^ cells validated Lag3-specific labeling (Fig. S1B).

To test whether Lag3 cycling also occurs in vivo, we intravenously administered PE-anti-Lag3 Ab into mice and Lag3 labeling was determined 2 and 4 h postinjection. Splenocytes were collected and stained for T cell subsets using CD4, CD8, CD25, and CD44 markers as described above. The results revealed Lag3 cycling analogous to those observed in vitro, with memory phenotype (CD44^high^) and Treg (CD25^+^) cells exhibiting increased Lag3^+^ cell frequencies at 2 and 4 h (Fig. 1D). Therefore, Lag3 cycling in memory phenotype and Treg cells also appears to occur in vivo.

Surface expression of co-inhibitory receptors is an activation-dependent process.^25^ Since Lag3 cycling was predominantly observed in CD44^high^ memory phenotype and Treg cells, we wondered whether it is driven by TCR engagement with MHC molecules. Anti-I-A^b^ mAb (Y3P) was added during the incubation to block TCR: MHCII interactions. We found that Lag3 cycling remained unchanged (Fig. S1C), suggesting that Lag3 cycle at steady state conditions does not require TCR interaction with MHCII. Taken together, these findings demonstrate that Lag3 undergoes constitutive cycling in T cells, with significantly higher levels observed in CD44^high^ memory and CD25^+^ Treg phenotype cells.

### Activated CD4 and CD8 T cells have distinct Lag3 cycling patterns

Activation of T cells rapidly upregulates surface Lag3 expression,^7^ and we next questioned if activation status alters Lag3 cycling kinetics. Naive CD4 or CD8 T cells were activated with immobilized anti-CD3 plus anti-CD28 mAbs without any exogenous cytokines. Cells were harvested 48 h later and then rested for 24 h in the presence of recombinant IL-2, expecting that the resting would diminish surface expression of Lag3 so that Lag3 cycling can be assessed (Fig. 2A). We first noted that activated CD8 T cells significantly upregulated surface Lag3 expression during 24 h of resting, whereas activated CD4 T cells downregulated surface Lag3 expression during the same resting period (Fig. 2B). Lag3 cycling of activated T cells was markedly different between the subsets. During 4-h incubation, Lag3 cycling was drastically increased in activated CD8 T cells, whereas activated CD4 cells exhibited a substantially lower Lag3 cycling (Fig. 2B and C). These findings indicate that Lag3 undergoes continuous post-activation cycling, with more robust kinetics in CD8 compared to CD4 T cells. We also examined whether T cells activated in vivo exhibit similar Lag3 cycling. We utilized experimental autoimmune encephalomyelitis (EAE), a mouse model of multiple sclerosis. CNS-infiltrating cells were harvested at the peak of the disease (day 18 post induction) and incubated with PE-conjugated anti-Lag3 antibody at 37 °C for 2 and 4 h. Similar to in vitro activated T cells, Lag3 labeling of CNS-infiltrating CD4 T cells continuously increased over time (Fig. 2D), demonstrating active Lag3 cycling in activated CD4 T cells in vivo.

**Figure 2:**
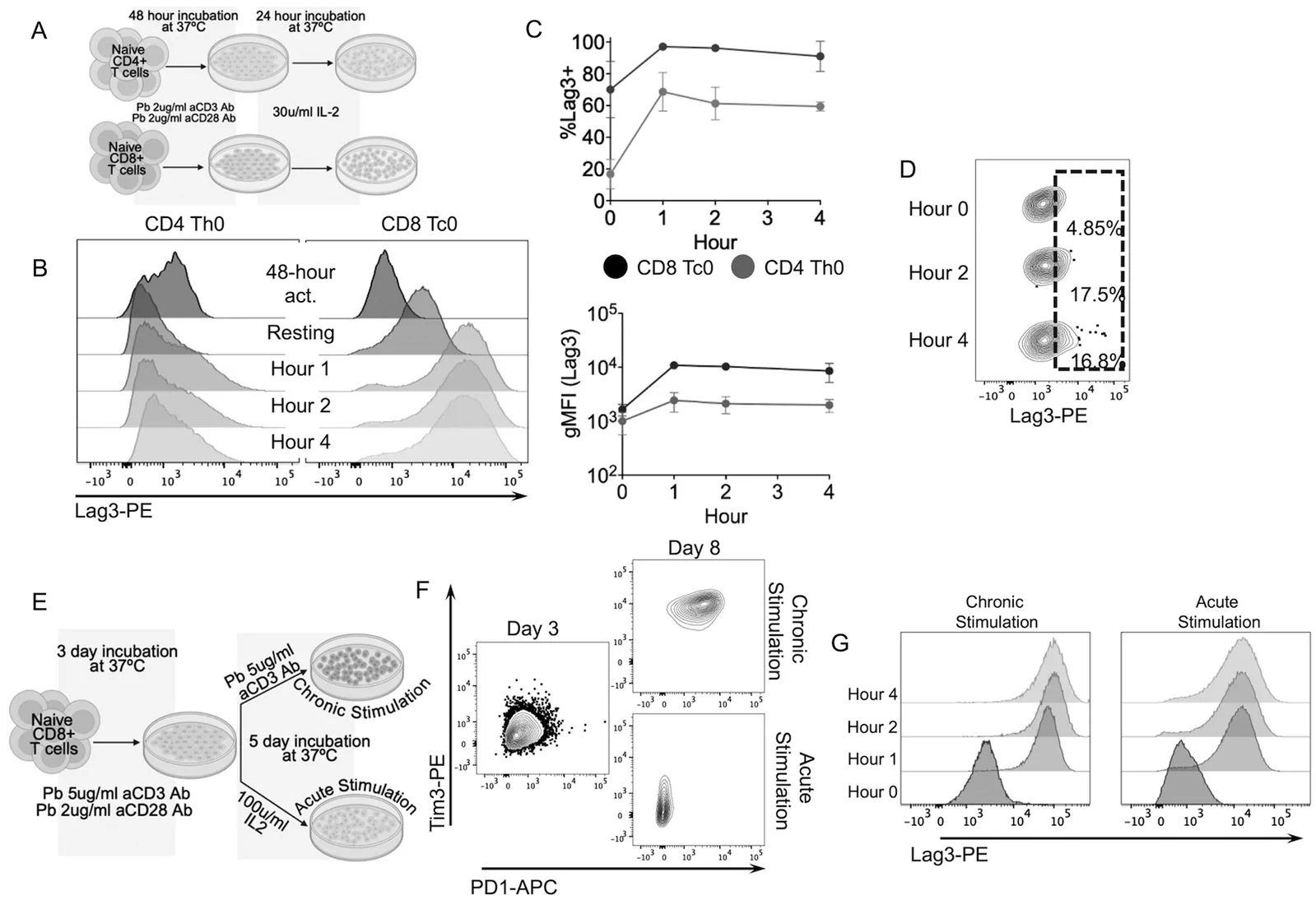
Activated CD4 and CD8 T cells have distinct Lag3 cycling patterns. (A) Schematic of in vitro T cell activation and resting protocol. Naive CD4 and CD8 T cells were activated with plate-bound anti-CD3 and anti-CD28 antibodies for 48 h, then rested for 24 h in the presence of IL-2. (B) Representative histoplots of Lag3 staining on CD4 and CD8 T cells after 48-hour activation, after 24-hour resting, and during 1-, 2-, and 4-h incubation with PE-conjugated anti-Lag3 antibody. (C) Quantification of Lag3 cycling in CD4 (gray) and CD8 (black) T cells over time. Data are shown as mean ± SEM from (*n*=3). (D) Lag3 cycling in CNS-infiltrating CD4 T cells isolated at the peak of EAE. Cells were incubated with PE-conjugated anti-Lag3 antibody for 2 or 4 at 37°C. Plot shows frequency mean for replicate (*n*=5). (E) Experimental schematic for generating exhausted versus non-exhausted CD8 T cells. Naive CD8 T cells were cultured under either chronic stimulation (exhausted) or acute stimulation followed by IL-2 culture (non-exhausted). (F) Flow cytometry validation of exhaustion status. Acutely stimulated CD8 T cells expressed low Tim3 and minimal PD1, whereas chronically stimulated cells displayed high levels of both Tim3 and PD1. (G) Lag3 cycling in exhausted and non-exhausted CD8 T cells measured by PE-conjugated anti-Lag3 antibody incubation at 37°C for 1–4 h. Data shown are a representative of three independent experiments.

Prolonged stimulation could cause T cell exhaustion, a state characterized by diminished effector function and sustained expression of inhibitory receptors including Lag3.^26^ To test whether Lag3 cycles in exhausted CD8 T cells, CD8 T cell exhaustion was induced in vitro as previously reported.^27^ T cells receiving chronic and acute stimulation as illustrated in Fig. 2E were stained for Tim3 and PD1 to validate their exhausted and non-exhausted states (Fig. 2F). Acutely stimulated T cells followed by 5-d incubation with IL-2 expressed low Tim3 and almost no PD1, whereas chronically stimulated “exhausted” T cells highly expressed both Tim3 and PD1 as previously reported.^27^ Incubation of these cells with PE-conjugated anti-Lag3 antibody at 37 °C revealed that surface Lag3 staining reaches a plateau within 1 h, and then the level remained high over 4-h period (Fig. 2G). Notably, exhausted CD8 T cells exhibit significantly higher levels of Lag3 expression compared to their non-exhausted counterparts (Fig. 2G), which is likely attributed to continuous TCR stimulation in chronically stimulated T cells. Therefore, higher Lag3 expression and cycling patterns are features of activated as well as exhausted T cells.

### Other co-inhibitory receptors also cycle in T cells

Given that CTLA4 preferentially cycle in Treg lineage cells^24^ and that Lag3 cycling is pronounced in memory and regulatory phenotype subsets, we next examined whether other co-inhibitory receptors, including CTLA4, PD1, and Tim3, also display the ability to cycle. Analogous to the experiments performed in Fig. 1, lymph node cells were incubated with fluorochrome-conjugated antibodies against PD1, CTLA4, and Tim3 for up to 4 h and receptor cycle was determined in CD44^high^ and CD44^low^ T cell subsets. First, preferential CTLA4 cycling in Treg lineage cells was confirmed (Fig. 3A), consistent with the previous report.^24^ We also found that CTLA4 cycling was substantially greater in memory phenotype CD4 T cells compared to that in CD8 T cells (Fig. 3A). PD1 cycling was particularly interesting. In brief, 50%–80% of Treg lineage cells became PD1^+^ after 4 h of incubation (Fig. 3B). Consistent with other receptors, memory phenotype CD4 T cells displayed the ability to extensively cycle PD1, as >50% became PD1^+^. Notably, naive CD4 T cells also cycled PD1, and the level was especially greater than other receptors (Fig. 3B). On the other hand, PD1 cycling of CD8 T cells was less evident compared to that of CD4 lineage cells (Fig. 3B). Although Tim3 cycling was less pronounced than that of other receptors, it still followed similar cycling pattern associated with Treg and memory phenotype T cell (Fig. 3C). These findings demonstrate that co-inhibitory receptors undergo cycling that is especially greater in Treg and memory phenotype T cells, with the dynamics and extent of cycling varying by receptors (Fig. 3D). Of note, PD1 expression in T cells is activation-induced, and naive T cells are known not to express PD1.^28^ Since we see significant PD1 cycling especially in CD44^low^ CD4 T cells (Fig. 3B), we examined whether it is a feature of true antigen-inexperienced naive T cells by utilizing TCR transgenic T cells. Transgenic TCR Vα/Vβ^+^ CD44^low^ 2D2 and P14 T cells were examined for PD1 cycling. PD1 cycling in these foreign antigen-specific naive T cells was minimal (Supp Fig. S2A), suggesting that TCR reactivity of polyclonal naive CD4 T cells against self-antigens may play a role in co-inhibitory receptor cycling (Fig. 3).

**Figure 3:**
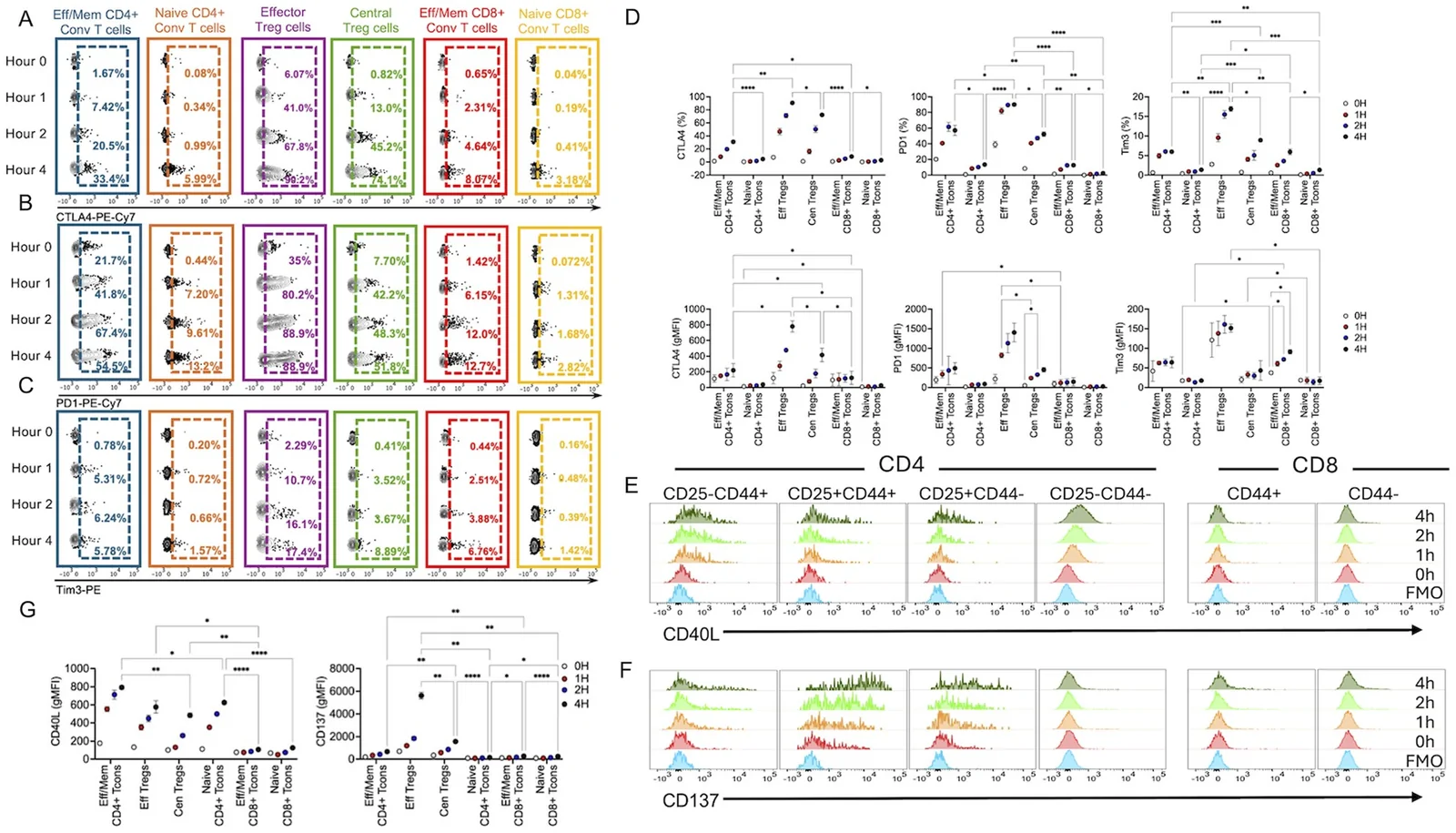
Co-inhibitory Receptor cycling in T cells. (A–C) Representative flow cytometry plots of total T cells incubated with fluorochrome-conjugated anti-PD1 (A), anti-CTLA4 (B), or anti-Tim3 (C) for the indicated times (0, 1, 2, and 4 h). Percentages denote receptor positive cells within the gate. Columns represent each T cell subset. Rows represent each timepoint. (D) Quantification of cycling over time for co-inhibitory receptors PD1, CTLA4 and Tim3 shown as % receptor positive cells (top row) and geometric mean fluorescence intensity (gMFI; bottom row) for each subset. Data are shown as mean ± SD of 3 independent experiments. (E, F) Representative flow cytometry plots of total T cells incubated with fluorochrome-conjugated anti-CD40L (E) or anti-CD137 (F) for the indicated times (0, 1, 2, and 4 h). Percentages denote receptor positive cells within the gate. Columns represent each T cell subset. Rows represent each timepoint. (G) Quantification of cycling over time for co-stimulatory receptors CD40L (left) and CD137 (right) shown as geometric mean fluorescence intensity (gMFI) for each subset. Data are shown as mean ± SD of 3 independent experiments. Statistical analysis was performed using 2-way repeated measures ANOVA with Geisser-Greenhouse correction. Tukey’s post hoc test was used for pairwise comparisons between time points within each population. *, *P* < 0.05; **, *P* < 0.01; ***, *P* < 0.001; ****, *P* < 0.0001.

We also investigated whether receptor cycling is a property unique to co-inhibitory receptor family or it rather represents a more general feature shared by costimulatory receptors. Since co-inhibitory receptors are largely activation-induced, we focused on activation-induced costimulatory receptors, such as CD40L and CD137 (4-1BB). Similar to co-inhibitory receptors, both costimulatory molecules exhibited preferential cycling in CD4 T cells with regulatory or effector/memory phenotypes (Fig. 3E and F). Interestingly, costimulatory receptor cycling was not observed in CD8 lineage T cells even within effector/memory subsets, suggesting that receptor cycling could be regulated by distinct mechanism in different T cell lineages.

### T cells cycling Lag3 also cycle PD-1

T cells with an exhaustion phenotype tend to express multiple co-inhibitory receptors.^26^^,^  ^29^ As memory phenotype and Treg cells display the ability to extensively cycle co-inhibitory receptors, we next examined whether T cells can cycle multiple receptors. Lymph node cells were incubated with both anti-PD1 and anti-Lag3 Abs and their cycling pattern was determined. We found that all Lag3-cycling memory phenotype and effector Treg cells also cycled PD-1, while not all PD-1 cycling cells cycled Lag3 (Fig. 4A and B). Interestingly, memory phenotype CD8^+^ T cells showed somewhat distinct cycling patterns between Lag3 and PD-1, as three distinct subsets were apparent: cells cycling Lag3 and/or PD-1 (Fig. 4A and B). Of note, PD-1 cycling patterns remained unchanged in resting Lag3-deficient T cells, suggesting that PD-1 cycling appears to be independent on Lag3 cycling (Fig. S2B). To measure Lag3 and PD-1 cycling in activated T cells, CD4 and CD8 T cells were activated for 3 d and subsequently rested for additional 24 h before examined for receptor cycling as described in Fig. 2A. Both activated T cell subsets extensively cycled both PD1 and Lag3, and the cycling of both receptors was more pronounced in activated CD8 T cells (Fig. 4C and D).

**Figure 4:**
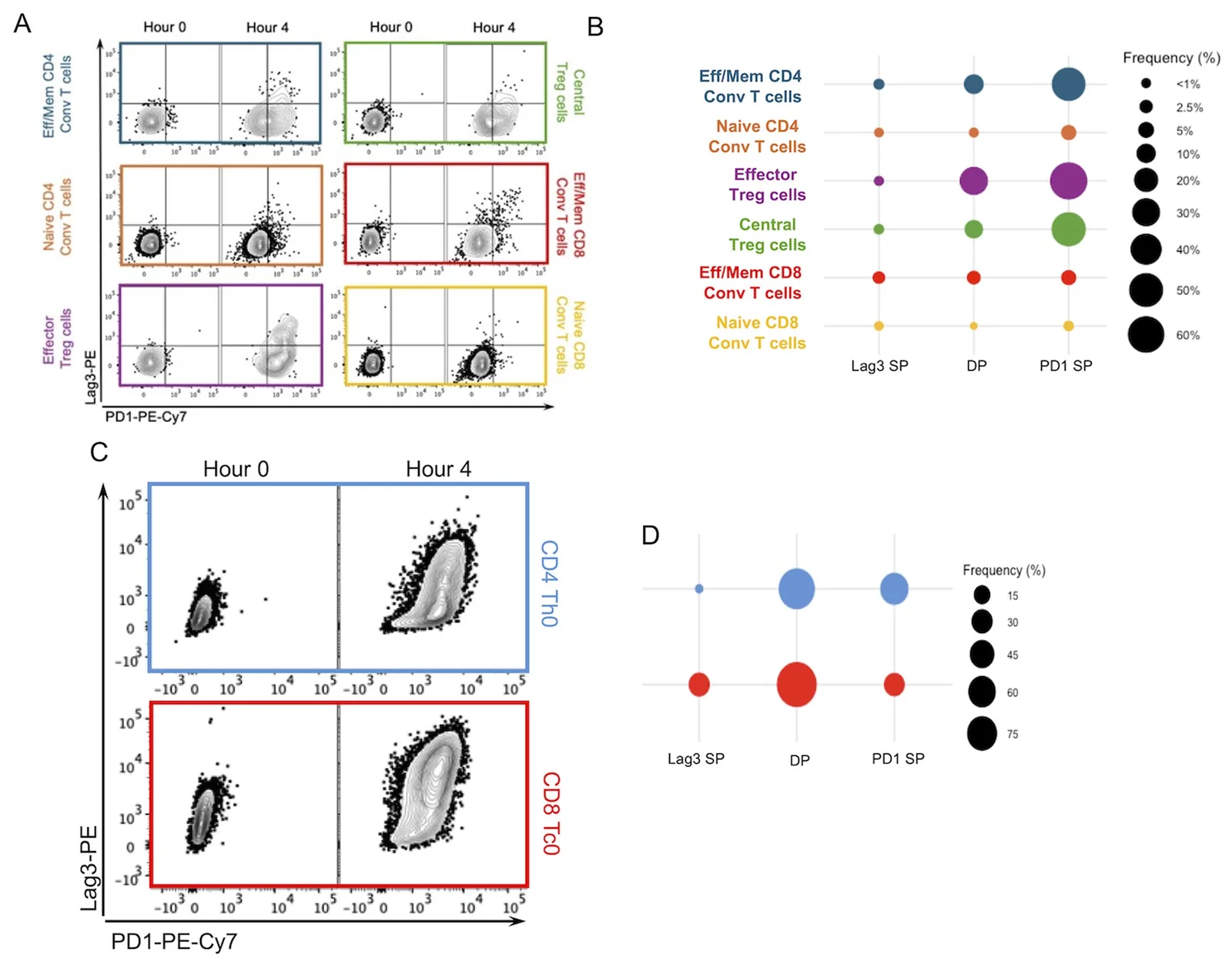
T cells cycling Lag3 also cycle PD-1. (A) Representative flow plots of PD-1 (*x*-axis; anti–PD-1-PE-Cy7) versus Lag3 (*y*-axis; anti-Lag3-PE) from total lymph node cells incubated with both antibodies at 37°C (cycling assay). Shown at hour 0 and 4 for the indicated subsets. (B) Dot matrix summarizing resting subsets at 4 h displayed as bubble plots; circle size encodes frequency (%) of Lag3 single positive (Lag3 SP), double-positive (DP), or PD1 single-positive (PD1 SP) cells, colored to match panel A. (C) Representative plots from activated cells. CD4 and CD8 T cells were activated for 2 d and rested 24 h before the cycling assay with both anti-PD1 and anti-Lag3 antibodies (as in Fig. 2A) at hour 0 and 4. (D) Dot matrix quantification for activated CD4 Th0 and CD8 Tc0 cells at 4 h (Lag3 SP, DP, PD1 SP; circle size = frequency), colored to match panel C. The experiments were repeated three times and similar results were observed.

### The endosomes are a critical organelle for Lag3 cycling

It was previously reported that intracellular Lag3 is largely found in the endosomes.^18^ To test whether the endosomes play a role in Lag3 cycling, activated T cells were treated with pronase without resting period to remove proteins on the cell surface. Following pronase treatment, cells were incubated with PE-anti-Lag3 Ab at either 16 °C or 37 °C (Fig. 5A). In brief, 16 °C incubation method was chosen since it allows internalization to occur while inhibiting endosome mediated trafficking.^30–34^ In CD4 Th0 cells, pronase effectively removed surface Lag3 and Lag3 cycling was drastically diminished at 16 °C compared to that seen at 37 °C incubation (Fig. 5B and C), suggesting important roles of the endosomes in Lag3 cycling. CD8 Tc0 cells showed similar patterns to the CD4 Th0 cells with decreased Lag3 cycling at 16 °C, and the extent of Lag3 cycling at 37 °C was substantially higher (Fig. 5B and C). Therefore, endosome-mediated cycling seems the key process of Lag3 cycling in activated T cells.

**Figure 5:**
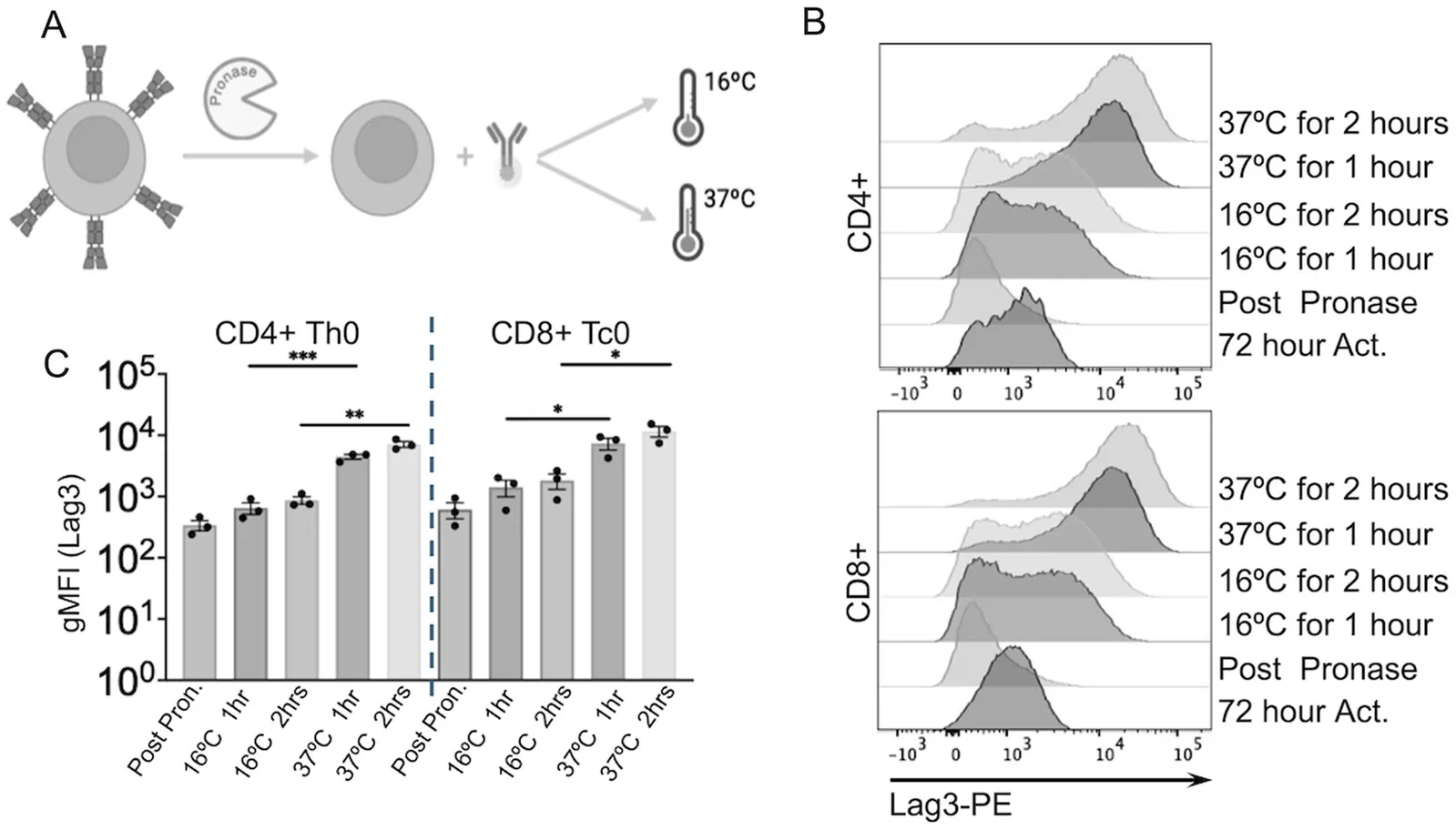
The endosomes are a critical organelle for Lag3 cycling. (A) Schematic of the assay. Activated T cells were treated with pronase to remove surface Lag3, then immediately incubated with PE–anti-Lag3 at either 16°C (inhibiting endosome-mediated recycling while permitting internalization) or 37°C (permits recycling). (B) Representative overlaid histoplots of Lag3-PE for CD4 Th0 (top) and CD8 Tc0 (bottom) cells at the indicated steps: baseline after 72 h activation, immediately post-pronase, and after 1 h or 2 h capture at 16°C or 37°C. (C) Quantification of Lag3 cycling as gMFI for CD4 Th0 and CD8 Tc0 cells under the same conditions. Data are shown as the mean ± SEM (*n*=3).

### Lag3 cycles independent of its cytoplasmic domain

CTLA-4 cycle requires tyrosine-based YVKM motif that interacts with AP-2 protein for cycling.^35^ Although the cytoplasmic domain of Lag3 does not contain such tyrosine-motifs, it consists of several highly conserved motifs, including the FxxL, KIEELE and EP repeats.^36^^,^^37^ These conserved motifs have been implicated in mediating Lag3-dependent T cell inhibition.^36^^,^^37^ We sought to determine if the cytoplasmic domain plays a role in Lag3 cycling. We recently reported a mutant animal model in which the entire Lag3 cytoplasmic domain is deleted.^38^ Lag3_CyD_-deficient (Lag$3_{\text{CyD}}^{-/-}$) cells were thus used to examine the role of Lag3 cytoplasmic domain in Lag3 cycling. Steady state Lag3 cycling revealed that the cytoplasmic domain seems to be dispensable (Fig. 6A). T cell subsets cycled Lag3 with similar kinetics to WT cells (Fig. 6B). We next activated these cells and saw no difference in surface expression after 48 hours of activation (Fig. 6C). Analogous to Fig. 2A, these cells were then rested and stained for cycling Lag3 and there was also no significant difference (Fig. 6D). This was unexpected, as for many receptors with well characterized cycling behavior, internalization is typically dependent on clathrin-mediated pathways which require proteins to bind to the receptor’s cytoplasmic domain in order to initiate the process.

**Figure 6:**
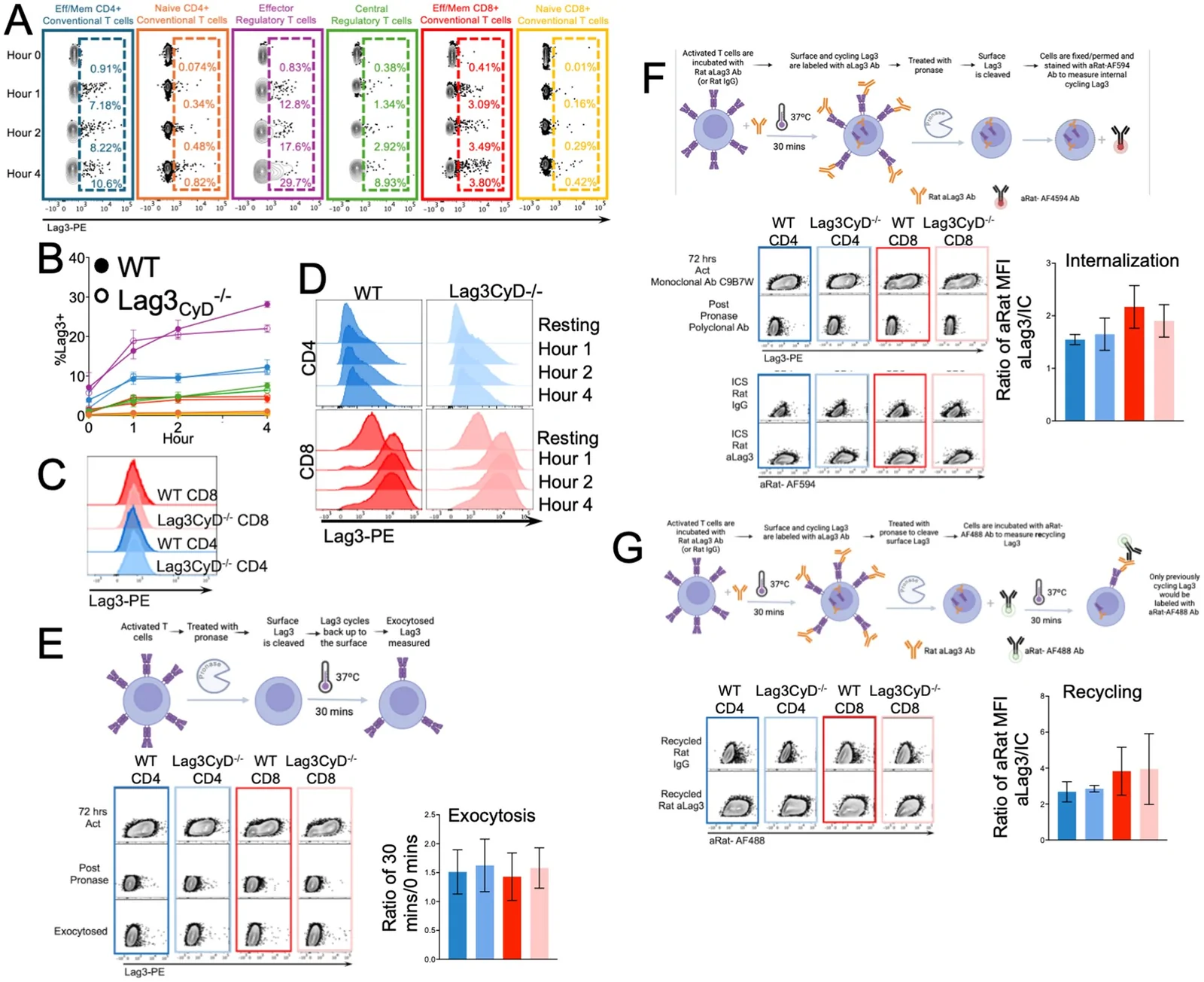
Lag3 cycles independent of its cytoplasmic domain. (A) Representative plots of Lag3 cycling in resting lymph node from T cell-specific Lag$3_{\text{CyD}}^{-/-}$ mice. Cells were incubated with PE–anti-Lag3 at 37°C and collected at 0, 1, 2, and 4 h. Percentages denote Lag3 positive cells of each T cell subset. (*n*=3) (B) Quantification of Lag3 cycling comparing wild type (WT, filled circles) and Lag$3_{\text{CyD}}^{-/-}$ (open circles) cells across T cell subsets. Data are shown as mean ± SEM (*n*=3). (C) Overlaid histoplots show surface Lag3 on activated CD4 (blue) and CD8 T cells (red) from both WT (dark) and Lag$3_{\text{CyD}}^{-/-}$ mice (light). (D) Overlaid histoplots show Lag3 cycling after a resting period in activated WT and Lag$3_{\text{CyD}}^{-/-}$ cells at timepoints of 0 (resting), 1, 2, and 4 h. Colored to match panel B. (E) Exocytosis assay. WT and Lag$3_{\text{CyD}}^{-/-}$ CD4 Th0 and CD8 Tc0 cells were activated for 72 h, treated with pronase, then allowed 30 min at 37°C for re-appearance of Lag3. Cells were then stained on ice with PE–anti-Lag3. First row represents plots of surface Lag3 after 72 hours activation. Second row represents surface Lag3 level after treatment with pronase. Third row represents surface Lag3 level after cells were incubated at 37°C for 30 mins and stained for Lag3 post incubation on ice. (F) Internalization assay. Cells were incubated for 30 min at 37°C with unconjugated rat anti-Lag3 mAb (C9B7W) to label surface Lag3, treated with pronase to remove residual surface antibody, fixed/permeabilized, and stained with AF594-conjugated anti-rat antibody. First row represents plots of surface Lag3 level after 72 hours of activation. Second row represents surface Lag3 level after treatment with pronase stained with polyclonal antibody. Third row represents cells incubated with rat IgG isotype antibody and later fixed/permeabilized and stained with AF594-conjugated anti-rat antibody. Fourth row represents cells incubated with rat anti-Lag3 antibody and later fixed/permeabilized and stained with AF594-conjugated anti-rat antibody. (G) Recycling assay. Activated cells were incubated for 30 min at 37°C with unconjugated rat anti-Lag3 mAb (C9B7W) to label surface Lag3, treated with pronase to remove residual surface antibody. The cultures were returned to 37°C for 30 min to allow recycling of internalized Lag3 to the surface, then recycled Lag3 was stained with AF488-conjugated anti-rat secondary on ice. First row represents cells incubated with rat IgG isotype antibody and later stained for recycled Lag3 with AF488-conjugated anti-rat secondary on ice. Second row represents cells incubated with rat anti-Lag3 antibody and later stained for recycled Lag3 with AF488-conjugated anti-rat secondary on ice Bar graphs show the mean ± SD (*n*=3). Colored to match subsets in Panel (D–F).

Due to Lag3’s unexpected deviation from canonical cycling mechanisms, we further examined its cycling by dissecting the process into distinct phases: exocytosis, internalization, and recycling. We defined exocytosis as the reappearance of Lag3 on the cell surface following pronase treatment. WT and Lag$3_{\text{CyD}}^{-/-}$ CD4 and CD8 T cells were activated for 72 h, treated with pronase to cleave surface Lag3 and then incubated at 37 °C for 30 mins to allow for surface reappearance of Lag3. Cells were then stained with PE-conjugated anti-Lag3 on ice (Fig. 6E). Although the reappearance of Lag3 was minimal during the incubation period, we found no significant difference between WT and Lag$3_{\text{CyD}}^{-/-}$ cells (Fig. 6E).

To assess internalization, activated cells were first incubated with an unconjugated rat anti-Lag3 mAb (C9B7W) at 37 °C for 30 minutes to allow surface Lag3 labeling and internalization. The cells were then treated with pronase to remove residual Ab-bound surface Lag3, leaving only internalized Lag3-antibody complexes. Cells were then fixed, permeabilized, and stained with a conjugated secondary anti-rat antibody to detect internalized Lag3 bound with rat anti-Lag3 mAb (Fig. 6F). Internalization was comparable between WT and Lag$3_{\text{CyD}}^{-/-}$ T cells (Fig. 6F).

Lastly, to evaluate recycling, we used the same cells from the internalization assay. After anti-Lag3 antibody bound Lag3 was internalized and surface Lag3 was removed via pronase, cells were incubated at 37 °C again for 30 min to allow for recycling of internalized Lag3 back to the surface. We then stained with a fluorescently conjugated anti-rat secondary antibody to detect recycled Lag3-mAb complexes at the surface (Fig. 6G). This assay confirmed that Lag3 recycles to the plasma membrane, with no significant difference between WT and Lag$3_{\text{CyD}}^{-/-}$ cells (Fig. 6G). Taken together, these findings indicate that the cytoplasmic domain of Lag3 is dispensable for cycling, implying that alternative mechanisms may regulate this process.

### Cells cycling Lag3 exhibit a survival and proliferation advantage in lymphopenic environments

To investigate the functional consequences of Lag3 cycling, we generated a novel cell type specific Lag3 overexpression mouse model (Lag3^OE^). The Lag3^OE^ model was generated by inserting a floxed transcriptional stop cassette upstream of the Lag3 cDNA at the *Rosa26* locus under the control of the CAG promoter (Fig. 7A). In the presence of Cre recombinase, the stop cassette is excised, resulting in constitutive expression of Lag3, irrespective of activation status (Fig. 7A). Upon crossing to CD4^Cre^ mice, CD4 and CD8 T cells from CD4^Cre^ Lag3^OE^ mouse expressed markedly higher levels of surface Lag3 compared to CD4^Cre^ (WT) controls (Fig. 7B). We then investigated whether constitutive expression alters the dynamics of Lag3 cycling. From utilizing Lag3^OE^ system, we were able to identify two distinct populations with different Lag3-cycling patterns (Fig. 7B), suggesting that there may be additional features regulated by Lag3 cycling. Of note, Lag3-noncycling T cells from Lag3^OE^ system still expressed higher surface Lag3 than that of WT mice (Fig. 7B). To determine whether cycling status impacts homeostatic expansion and fitness, we FACS sorted Lag3-cycling and Lag3-noncycling naive CD4 and CD8 T cells from CD4^Cre^ Lag3^OE^ mice, labeled with CFSE and adoptively transferred into sublethally irradiated WT recipients (Fig. 7C). Fourteen days later, analysis of CFSE dilution revealed that Lag3-cycling T cells proliferated more than Lag3-noncycling cells (Fig. 3D and E). The recovery from the secondary lymphoid tissues of transferred Lag3-cycling cells was greater than Lag3-noncycling cells in both CD4 and CD8 subsets, although the difference did not reach statistical significance (Fig. 7E). Therefore, Lag3-cycling status appears to alter homeostatic proliferation and survival advantage over their noncycling counterparts.

**Figure 7:**
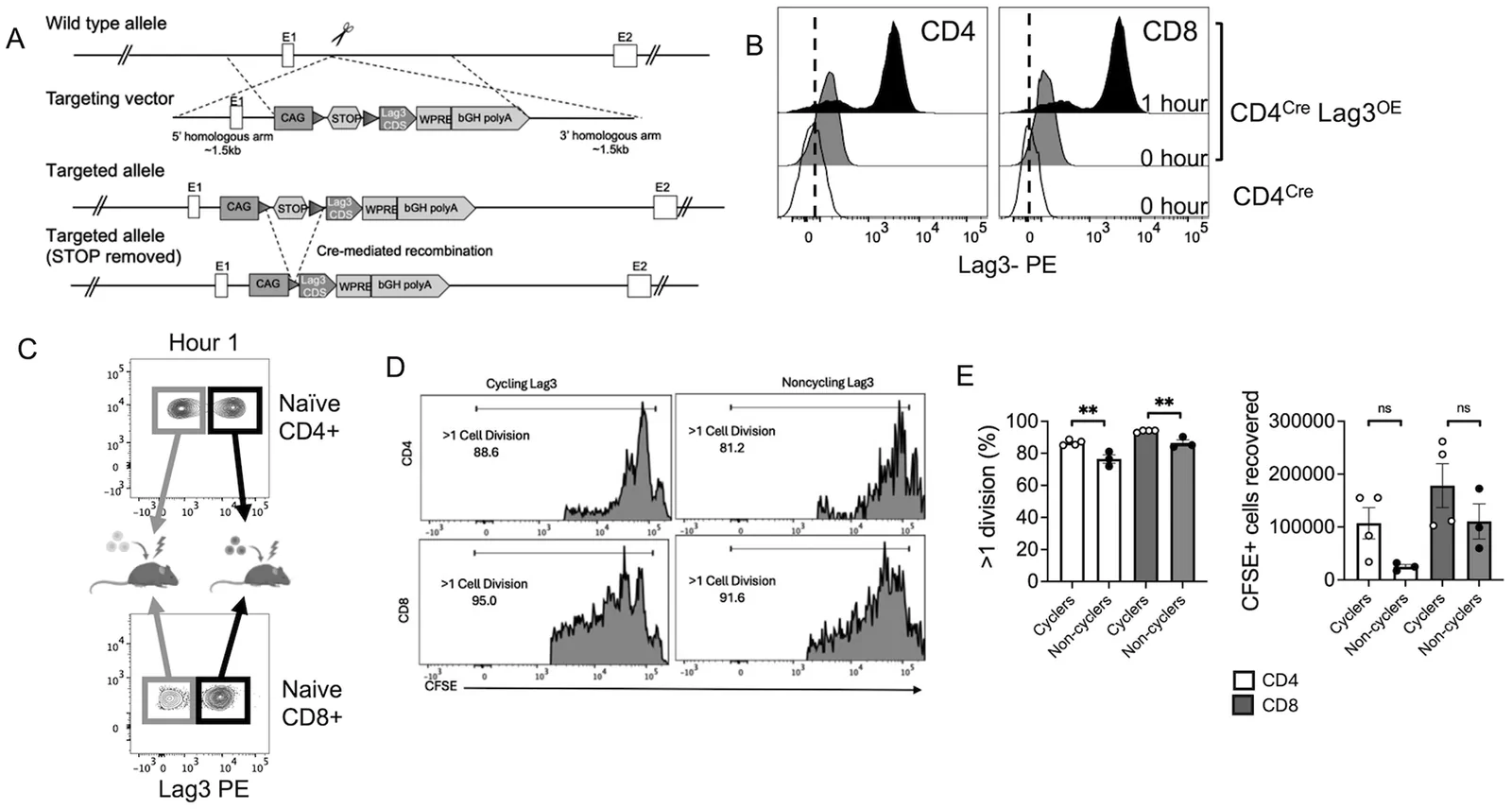
Lag3^OE^ cells cycling Lag3 exhibit advantages in proliferation within lymphopenic environments. (A) Generation of the conditional Lag3 overexpression mutant mouse (Lag3^OE^). Schematic showing the targeting strategy: a floxed transcriptional STOP cassette upstream of Lag3 cDNA was inserted at the *Rosa26* locus under the CAG promoter. Cre-mediated recombination excises the STOP cassette, enabling constitutive Lag3 expression in T cells. (B) Validation of the model. Representative histoplots of cycling Lag3 (1 h) and surface Lag3 (0 h) expression on CD4 (right) and CD8 (left) T cells from CD4^Cre^ Lag3^OE^ mice versus control (CD4^Cre^). (C) Cycling and noncycling naive CD4 and naive CD8 T cells were identified and sorted from CD4^Cre^ Lag3^OE^ mice, CFSE-labeled, and adoptively transferred into sublethally irradiated WT recipients. (D) Representative plots gated on >1 divisions based on CFSE dilution of donor cycling and non-cycling CD4 and CD8 T cells after 14 d post transfer. (E) Frequency of cells with >1 division and the absolute numbers of CFSE^+^ from total cycling and noncycling CD4 and CD8 donor T cells recovered from lymph nodes (white bars: CD4 cells, grey bars: CD8 cells) (*n*=3–4) **P<0.01.

### Lag3-cycling cells exhibit proliferative and survival advantages in steady-state condition

To further validate the role of Lag3-cycling in T cell homeostasis at steady-state condition, we compared proliferation potential between Lag3-cycling and Lag3-noncycling naive or memory, and CD4 or CD8 T cells from wild type mice. Ki-67 expression was markedly increased in memory phenotype T cells, consistent with the literature that CD44^high^ cells are actively proliferating cells.^39^ While Lag3-cycling status of CD44^high^ cells drastically increased Ki-67 expression, that of naïve subsets did not support Ki-67 expression (Fig. 8A). We then measured the expression for homeostatic cytokine receptors, ie CD127 and CD122, in these cells. Unexpectedly, CD127 expression appeared to be higher in Lag3-noncycling T cells, whereas CD122 expression was greater in Lag3-cycling cells, except memory phenotype CD8 T cells (Fig. 8B). Nevertheless, IL-7 responsiveness measured by IL-7-induced pSTAT5 expression was significantly elevated in Lag3-cycling CD4 (Fig. 8C) and CD8 (data not shown) T cells. Furthermore, Bcl2 expression was also elevated in Lag3-cycling CD4 T cells (Fig. 8D) as well as in CD8 T cells (data not shown). These findings suggest that Lag3-cycling T cells may exhibit increased sensitivity to the homeostatic cytokine IL-7, independent of the receptor expression, thereby promoting enhanced homeostatic expansion in vivo.

**Figure 8:**
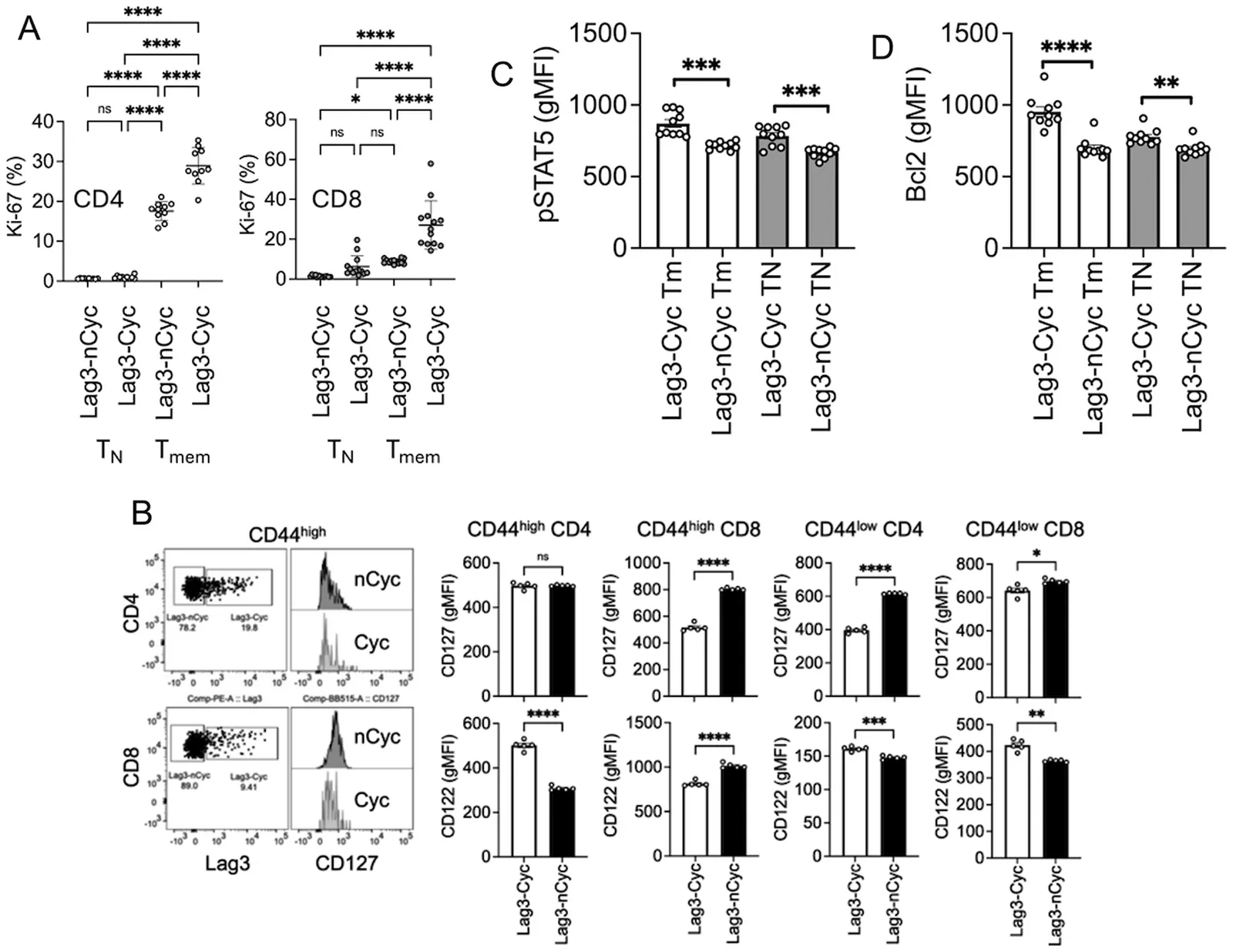
Lag3-cycling T cells display enhanced proliferative and survival capacity at steady state. (A) Ki-67 expression in Lag3-cycling (Cyc) versus non-cycling (nCyc) T cells. Left: CD4^+^ T cells; right: CD8^+^ T cells. Naive (T_N_) and memory (T_mem_) subsets were separately analyzed. Data are shown as individual points with mean ± SEM. *P<0.05; ****P<0.0001. (B) Homeostatic cytokine receptor expression in Lag3-cycling (Cyc) versus noncycling (nCyc) T cells. Left: representative flow cytometry plots showing Lag3 versus CD127 staining in CD44^high^ CD4^+^ and CD8^+^ T cells, with Lag3-cycling (Cyc) and non-cycling (nCyc) gates indicated. Right: quantification of CD127 (top row) and CD122 (bottom row) gMFI in Lag3-cycling versus noncycling cells across CD44^high^ CD4^+^, CD44^high^ CD8^+^, CD44^low^ CD4^+^, and CD44^low^ CD8^+^ subsets. *, *P*<0.05; **, *P*<0.01; ***, *P*<0.001; ****, *P*<0.0001. (C) Lag3-cycling and non-cycling memory (T_mem_) and naive (T_N_) CD4^+^ T cells were stimulated with IL-7, and pSTAT5 gMFI was quantified. ***, *P*<0.001. (D) Bcl2 gMFI in Lag3-cycling versus non-cycling memory and naïve CD4^+^ T cells. **, *P*<0.01; ****, *P*<0.0001.

## Discussion

T cells with exhausted or regulatory properties are characterized by constitutive expression of co-inhibitory receptors, including CTLA4 and Lag3. In naive conventional T cells, CTLA4 and Lag3 are predominantly localized within intracellular endosomal compartments rather than at the plasma membrane, where they can be rapidly mobilized following activation. While these dynamics have been observed, our understanding the mechanisms that regulate the balance between intracellular sequestration, recycling, and activation-induced surface expression remain poorly defined. In this study, we demonstrate that Lag3 expression is not static but rather undergoes continuous intracellular cycling under homeostatic conditions. We show that this cycling pattern is strongly dependent on the differentiation status of the T cell, affected by Foxp3 and CD44 expression (ie regulatory T cells and memory/effector phenotype cells), suggesting that lineage-specific programs may actively control the dynamics of Lag3 expression. Other co-inhibitory and co-stimulatory receptors such as CTLA4, PD1, CD40L, and CD137, also exhibit similar lineage-specific cycling patterns. Unlike CTLA4 and PD1 that utilize intracellular motifs for constitutive internalization,^11^^,^^40^ we found that Lag3 internalization and recycling are not dependent on the cytoplasmic domain. Moreover, we found that Lag3-cycling T cells exhibit proliferation advantage during homeostatic proliferation and under state-state condition. Therefore, these results reveal a previously unknown Lag3 expression dynamics by which T cells may fine-tune their responsiveness to activation and homeostatic cues.

Under steady-state conditions, Lag3 expression on conventional resting T cells is minimal and largely induced following activation, a pattern similarly found for CTLA4. On the other hand, Treg cells constitutively express both Lag3 and CTLA4.^41^^,^^42^ Since CTLA4 expression has been previously shown to undergo continuous cycling, with markedly higher levels in Treg cells,^16^^,^^40^^,^^43^ we reasoned that Lag3 might follow a similar pattern. Indeed, the expression of CD44, a marker of memory/effector phenotype, and of CD25 was closely associated with the cycling dynamics of Lag3 as well as other co-inhibitory and co-stimulatory receptors, suggesting that this process may reflect a more generalized regulatory mechanism. The mechanistic basis of this cycling process remains to be clarified. One possibility is that it is linked to proliferative capacity, as both CD44^high^ cells and Treg cells are highly proliferating cells. Supporting this, activated T cells display greater Lag3 cycling, with CD8 T cells exhibiting faster and more extensive cycling as well as better surface retention compared to CD4 T cells, consistent with the fact that CD8 T cells undergo more rapid clonal expansion than CD4 T cells.^44^ Moreover, in vitro models of chronically stimulated exhausted CD8 T cells display even greater Lag3 expression and cycling, raising the possibility that cycling dynamics may be further amplified under conditions of sustained antigen exposure.

Wakamatsu et al. recently reported that Lag3-induced MHCII trogocytosis can be a mechanism indirectly suppressing CD4 T cell activation during cognate antigen-induced T cell-APC interaction,^45^ suggesting that Lag3 cycling observed in this study could also contribute to downregulation of T cell activation. However, given that Lag3 binding to stable peptide-MHCII complex is needed to limit T cell activation^46^ and that Lag3 cycling in effector/memory CD8 T cells is also efficient, we speculate that Lag3-mediatead MHCII trogocytosis may not occur during spontaneous receptor cycling without cognate antigen-dependent T cell-APC interaction. The fact that blocking MHCII molecule using anti-MHCII antibody doesn’t diminish Lag3 cycling further supports the possibility. Further investigation is needed to test the association between Lag3-cycling and Lag3-dependent regulation of T cell activity.

The molecular mechanism underlying PD1 and CTLA4 internalization has previously been examined. CTLA4’s cytoplasmic localization YVKM motif mediates binding to the clathrin adaptor protein, AP-2, promoting receptor internalization^47^^,^^48^ and disrupting the AP-2 binding site results in increased surface retention of the receptor.^13^ Expression of the motif in co-stimulatory CD28 receptor is sufficient to confer intracellular localization.^49^ On the other hand, the mechanisms regulating PD1 recycling remain incompletely understood. Previous work demonstrated that palmitoylation of the PD1 cytoplasmic domain promotes its interaction with Rab11, a key regulator of protein trafficking to the recycling endosome, thereby protecting PD1 from lysosomal degradation.^50^ In addition, Fut8-mediated glycosylation of the extracellular IgV domain is critical for PD1 surface expression, as genetic ablation of the Fut8 reduces PD1 surface expression and enhances T cell activation.^11^ Whether PD1 internalization occurs through clathrin-mediated endocytosis is still unclear. Notably, the transcription factor Tox, a central regulator of T cell exhaustion, has been implicated in driving PD1 recycling.^51^ Conversely, F-box protein 38 (FBXO38), an E3 ubiquitin ligase, targets PD1 for proteasomal degradation via K48 polyubiquitination.^52^ Because FBXO38 expression is downregulated in tumor infiltrating lymphocytes, PD1 degradation is attenuated.^53^ Given that we observed PD1 to exhibit the highest rate of cycling, further studies are required to define the precise molecular mechanisms governing its recycling. On the other hand, Lag3 presents a distinct case, as it lacks both tyrosine-based inhibitory motifs and the YVKM motif responsible for intracellular localization. Although 3 conserved motifs located in the Lag3 cytoplasmic domain have been implicated in its inhibitory functions,^2^^,^  ^20^^,^  ^36^^,^^37^^,^  ^54^ their role in regulating Lag3 internalization and recycling has not been established. Our findings that Lag3 cytoplasmic domain deficient cells exhibit unaltered internalization and recycling suggest that the mechanisms driving Lag3 trafficking are distinct from those employed by other inhibitory receptors.

Lag3 is constitutively cleaved by the metalloprotease ADAM10, with cleavage further enhanced by ADAM17 upon T cell activation.^9^ This shedding event provides an additional layer of regulation for the surface expression of Lag3. Although soluble Lag3 monomers generated through ADAM-mediated shedding appear to have little biological activity, an elegant study by Vignali and colleague using a non-cleavable Lag3 mouse model revealed that Lag3 shedding could be critical for mounting effective anti-tumor immunity.^55^ Our current studies did not directly address the contribution of ADAM-mediated cleavage to Lag3 cycling, raising the possibility that internalization, recycling, and shedding may be interrelated processes. Defining the relationship will be important for understanding how Lag3 expression is dynamically controlled.

Of note, Lag3 cycling is confined to a distinct subset of T cells, with some actively cycling Lag3 while others remain non-cycling. This heterogeneity became even more apparent in a model of constitutive Lag3 overexpression. When they were transferred into mildly lymphopenic hosts to induce self- and microbiome-dependent homeostatic proliferation, Lag3-cycling T cells exhibited greater proliferative capacity. The recovery of Lag3-cycling cells from the secondary lymphoid tissues was consistently and moderately increased, suggesting possible differences in in vivo trafficking and/or to susceptibility to cell death. At the cellular level, receptor cycling may control the protein composition of the plasma membrane, thereby regulating how cells interact with their environments and activation cues. Consistent with the notion, homeostatic fitness displayed by Lag3-cycling T cells was further confirmed in conventional wild type cells. Lag3-cyclilng CD4 and CD8 T cells exhibited increased proliferation as well as survival measured by Ki-67 and Bcl2 expression, respectively. Although homeostatic cytokine receptor expression, especially for IL-7, did not match with these advantages, Lag3-cycling cells expressed significantly enhanced pSTAT5 expression in response to IL-7 stimulation. Therefore, Lag3 cycling may influence cellular fitness for activation and homeostasis, although the precise impact of this process on T cell responses remains to be determined.

In conclusion, our findings identify Lag3 cycling as a previously unrecognized regulatory mechanism in T cells. Future investigation of the cellular and molecular pathways controlling this process will advance our understanding of co-inhibitory receptor biology and may uncover novel therapeutic strategies for conditions in which immune modulation by co-inhibitory receptors is critical, such as cancer and chronic inflammatory diseases.

## Supplementary Material

vkag039_Supplementary_Data

## Data Availability

The data underlying this article will be shared on reasonable request to the corresponding author.
