# Supplementary material for: Activation and lineage specific Lag3 expression dynamics in T cells
Source: J Immunol. 2026 Mar 28;215(3):vkag039. doi: 10.1093/jimmun/vkag039 (PMC13556269; doi:10.1093/jimmun/vkag039)
Supplement: Supplementary file 1 [file vkag039_supplementary_data.zip › Khaled et al. Supp Figures A1.docx]

**Activation and lineage specific Lag3 expression dynamics in T cells**

**Chaimae Khaled^1^, Sohee Kim^1^, Dongkyun Kim^1,2^, Booki Min^1,2,*^**

**^1^Department of Microbiology and Immunology, Northwestern University Feinberg School of Medicine, Chicago, IL 60611. USA**

**^2^Center for Human Immunobiology, Northwestern University Feinberg School of Medicine, Chicago, IL 60611. USA**

**Supplementary Figures:**

**Supplementary Figure S1. Validation of Lag3 cycling specificity and TCR-independence**

**(A)** Upper panel shows Lag3 gMFI, and lower panel shows the frequency of Lag3^+^ cells (%). Data represent mean ± SEM (n = 3). Statistical analysis was performed using two-way repeated measures ANOVA with Geisser-Greenhouse correction. Tukey's post-hoc test was used for pairwise comparisons between time points within each population. significant P values are shown. *, p<0.05; **, p<0.01; ***, p<0.001; ****, p<0.0001. **(B)** Representative plot for Lag3 cycling validation using Lag3^-/-^ cells. Lymph node cells from germline Lag3-deficient mice were subjected to the cycling assay, confirming antibody specificity. **(C)** TCR-MHCII interaction is dispensable for Lag3 cycling. Cells were pre-treated with anti-I-A^b^ blocking antibody (Y3P) or left untreated (control) prior to the cycling assay. Representative plots for CD44^high^ CD4⁺ T cells.

**Supplementary Figure S2. PD1 cycling in TCR transgenic T cells and Lag3-deficient cells**

**(A)**  Top: gating strategy for transgenic TCR Vα/Vβ⁺ CD44^low^ T cells from 2D2 and P14 TCR transgenic mice. Bottom: quantification of PD1⁺ frequency over time in 2D2 and P14 CD44^low^ naïve T cells at 0-, 1-, 2- and 3-hours incubation. **(B)**  Representative plot comparing PD1 cycling in WT versus Lag3^-/-^ CD44^high^ CD4⁺ T cells at 0, 1, 2, and 4 hours.
